# Promising effects of 33 to 36 Fr. bougie calibration for laparoscopic sleeve gastrectomy: a systematic review and network meta-analysis

**DOI:** 10.1038/s41598-021-94716-1

**Published:** 2021-07-26

**Authors:** Po-Chih Chang, Kai-Hua Chen, Hong-Jie Jhou, Po-Huang Chen, Chih-Kun Huang, Cho-Hao Lee, Ting-Wei Chang

**Affiliations:** 1 Division of Thoracic Surgery, Department of Surgery, Kaohsiung Medical University Hospital/Kaohsiung Medical University, Kaohsiung City, Taiwan; 2 Weight Management Center, Kaohsiung Medical University Hospital/Kaohsiung Medical University, Kaohsiung City, Taiwan; 3 Ph.D. Program in Biomedical Engineering, College of Medicine, Kaohsiung Medical University, Kaohsiung City, Taiwan; 4 Department of Sports Medicine, College of Medicine, Kaohsiung Medical University, Kaohsiung City, Taiwan; 5 Department of Surgery, Kaohsiung Medical University Hospital/Kaohsiung Medical University, No. 100, Tzyou 1st Road, Kaohsiung City, 80756 Taiwan; 6 Department of Neurology, Changhua Christian Hospital, Changhua, Taiwan; 7 School of Medicine, Kaohsiung Medical University, Kaohsiung, Taiwan; 8 Department of Internal Medicine, Tri-Service General Hospital, National Defense Medical Center, Taipei, Taiwan; 9 Body Science and Metabolic Disorders International Medical Center, China Medical University Hospital, Taichung City, Taiwan; 10 Division of Hematology and Oncology Medicine, Department of Internal Medicine, Tri-Service General Hospital, National Defense Medical Center, Taipei, Taiwan

**Keywords:** Obesity, Stomach, Weight management

## Abstract

The standard size of bougie for laparoscopic sleeve gastrectomy (LSG) is not yet established. Therefore, a systematic review and network meta-analysis were conducted to assess the weight loss effects and associated complications of LSG for patients with morbid obesity, based on different bougie sizes. A total of 15 studies were reviewed in this systemic review and network meta-analysis (2,848 participants), including RCTs and retrospective studies in PubMed, and Embase until September 1, 2020. The effectiveness of different bougie calibration sizes was assessed based on excess weight loss (EWL), total complications, and staple line leak. Within this network meta-analysis, S-sized (≤ 32 Fr.) and M-sized (33–36 Fr.) bougies had similar effects and were associated with the highest EWL improvement among all different bougie sizes (S-sized: standardized mean difference [SMD], 10.52; 95% confidence interval [CI] − 5.59 to  − 26.63, surface under the cumulative ranking curve [SUCRA], 0.78; and M-sized: SMD, 10.16; 95% CI − 3.04–23.37; SUCRA, 0.75). M-sized bougie was associated with the lowest incidence of total complications (M-sized: odds ratio, 0.43; 95% CI, 0.16–1.11; SUCRA, 0.92). Based on our network meta-analysis, using M-sized bougie (33–36 Fr.) is an optimal choice to balance the effectiveness and perioperative safety of LSG in the clinical practice.

## Introduction

Obesity has been a global endemic with a continuously increasing prevalence. Approximately > 650 million adults are obese in 2016, and thus, worldwide obesity has nearly tripled since 1975^1^. For its clinical significance due to comorbidities and associated economic impact, obesity therapies remain very diverse, from basic lifestyle management, diet counseling, physical exercise, medications, endoscopic interventions to invasive bariatric surgical procedures^1–3^. Among these therapies, bariatric surgery has been proven as the most effective therapeutic modality for morbid obesity due to its significant weight loss and long-term durability^4,5^.

With its surgical simplicity, no bowel manipulation/anastomosis, and relatively comparable weight loss effect, laparoscopic sleeve gastrectomy (LSG) has gained its popularity since 2003 and becomes the widely preferred bariatric surgical procedures for years^4,6–8^. Approximately 609,897 primary bariatric surgical interventions were performed worldwide in 2016, with LSG as the most commonly performed primary bariatric surgical procedure comprising 340,550 (53.6%) of patients, followed by Roux-en-Y gastric bypass (N = 191,326; 30.1%) and one-anastomosis gastric bypass (N = 30,563; 4.8%)^7^. The clinical significance of mastering intraoperative manipulation and associated surgical strategies during LSG could not be overemphasized for our modern bariatric surgeons.

Not only changes in gastric motility and related hormonal secretion but also dominant mechanisms of gastric restriction, based on the essential intraoperative bougie calibration with longitudinal gastric transection of the fundus, body, and antrum along the lesser curvature, lead to limited eating volume of about 100 mL, subsequent dietary habit modification, and eventually weight reduction^9,10^. Although the surgical standardization and relative contraindications for LSG have been well documented, the standard bougie size used to calibrate the gastric sleeve still remains to be established, based on the weight loss effects and related complications, such as staple line leak (SLL), gastric stenosis, or de novo reflux esophagitis^11–26^.

Although several systemic meta-analyzes, based on pooled data from case series and randomized controlled trials (RCTs), have been published, the standard bougie size for LSG is not yet established^16,17,21,23–28^. Herein, we performed a network meta-analysis and cataloged results of these controlled trials into a comprehensive systematic review and meta-analysis of available data to determine the standard bougie size for calibration during LSG, based on excess weight loss (EWL), associated complications, and SLL percentage.

## Materials and methods

Current network meta-analysis was performed after establishing guidelines from the Preferred Reporting Items for Systematic Reviews and Meta-Analyses extension for Network Meta-Analyses (PRISMA-NMA)^29,30^ and Meta-analyzes Of Observational Studies in Epidemiology (Table S1.1 and Table S1.2)^31^. Registered protocol was available in the Open Science Framework (https://osf.io/drhsb).

### Data sources and search strategy

A systematic publication review without language restrictions was performed and retrieved from PubMed and Embase from inception until September 1, 2020. Gray literature and manual searches for potentially eligible articles from review articles were reviewed. The US Government Clinical Trials database (www.ClinicalTrials.gov) was searched for ongoing clinical trials. The search terms comprised “laparoscopic sleeve gastrectomy,” “bougie calibration,” and “bougie size,” along with a list of all interventions and possibly relevant keywords. (Table S2).

### Inclusion and exclusion criteria

Both RCTs and observational cohort studies were included in the study design. Targets of comparison were patients with morbid obesity (aged at least 18 years) received LSG with bougie calibration to create the neogastric tube. Morbid obesity is defined as body mass index (BMI) of ≥ 40 kg/m^2^ or 35 kg/m^2^ generally associated with comorbidities, except for lower BMIs among East Asians^20,32^. The comparison included two or more different bougie sizes; studies reporting the relationship between bougie sizes and weight loss were enrolled in this study.

The exclusion criteria included (1) studies that evaluated adolescents or pediatrics participants, (2) those in which interested outcomes were not reported, (3) those not specific to patients with morbid obesity, and (4) single-arm studies without comparators.

Two authors (KH Chen, TW Chang) independently selected trials that met the inclusion criteria, and another author (KH Chen) adjudicated differences. In case of disagreement, the same authors consulted with another one (PC Chang) to achieve decisions after a deliberate group discussion.

### Data extraction and bias assessment

Two reviewers (HJ Jhou, TW Chang) independently screened the studies, extracted relevant data, and assessed the risk of bias among included studies using the Cochrane risk of bias tool (Table S3.1 and Table S3.2)^33^. Data extraction was performed using a special designed sheet obtained from reports of a previous meta-analysis^28^. Study information regarding studies, participants, and treatment characteristics was obtained. If available data are lacking, corresponding authors were contacted for data collection.

### Outcome definition

1. Percentage of EWL (% EWL): (weight loss/baseline excess weight) × 100, where weight loss = preoperative weight –the initial weight; baseline excess weight = initial weight − ideal weight (X) where X was calculated using an ideal BMI, and the ideal BMI cutoff point has been used differently in enrolled studies (Table S4).
2. Overall complications: All complications related to LSG, such as de novo gastroesophageal reflux disease (GERD), postoperative bleeding, nonspecific abdominal pain, nausea, dehydration, surgical site infection, portal vein thrombosis, or as defined by the study authors, were obtained (Table S4).
3. SLL: Postoperative neogastric tube leak or as defined by study authors (Table S4).

### Data synthesis and statistical analysis

Herein, study patients were classified into four different groups according to different ranges of calibrating bougie size: XL (extra-large, defined as the bougie size of > 40 Fr.), L (large, defined as the bougie size of 36–40 Fr.), M (median, defined as the bougie size is between 33 and 36 Fr., including 36 Fr.), and S (small, defined as the bougie size of < 32 Fr.).

We used the frequentist network meta-analysis (NMA) model to compare effect sizes among studies with the same interventions. All frequentist approach network meta-analyzes were performed using the statistical package Netmeta (Version 1.2-1)^34,35^ in R Project 3.6.1 (R Core Team, Vienna, Austria) and Stata version 16 (Stata Corp, College Station, Texas). The symmetry and geometry of the evidence were examined by producing a network plot with nodes for the number of study participants and connection sizes corresponding to the number of studies^36^. For continuous data, summary standardized mean differences (SMDs) with 95% confidence intervals (CIs) were calculated using a random-effects model (21) as a conservative estimate. For categorical data, summary odds ratios (ORs) with 95% CIs were estimated with 0.5 zero-cell correction^37^. All comparisons were set as two-tailed, and a *p* value statistical significant cutoff point was set at 0.05. For generating high resolution figure, we applied Adobe Photoshop CC 2018 and Sketch Version 45.1 software.

The rank of treatment within defined groups measured was measured using the surface under the cumulative ranking curve (SUCRA)^38^, which is the relative percentage of probabilities as the best treatment, in a scale from 0 (worst) to 1 (best)^39^. SUCRA can be clinically used to compare treatment effects of all treatments for the target outcomes.

Potential inconsistencies between direct and indirect evidences were compared within the network model. Moreover, global inconsistencies were examined using a design-by-treatment interaction model, whereas local inconsistencies between the included comparators were examined using a node-splitting method^40^. Statistical significance was set at 5% for analyzes. If the inconsistency existed, sensitivity analysis was performed to determine possible reasons.

The assumption of network transitivity was examined by visually inspecting tables with patient’s population across included studies, study methodologies, design intervention details, and outcome measurement differences^41^.

Comparison-adjusted funnel plots and Egger’s test regression were used to assess possible publication bias or potential small study effects for available interventions^42^.

### Ethical statement

There was no human trial in this systematic review and network meta-analysis, and the approval from the ethics committee does not apply.

### Consent statement

Because this study is a systematic review with network meta-analysis, the informed consent does not apply.

## Results

### Systematic literature review

**Figure 1:**
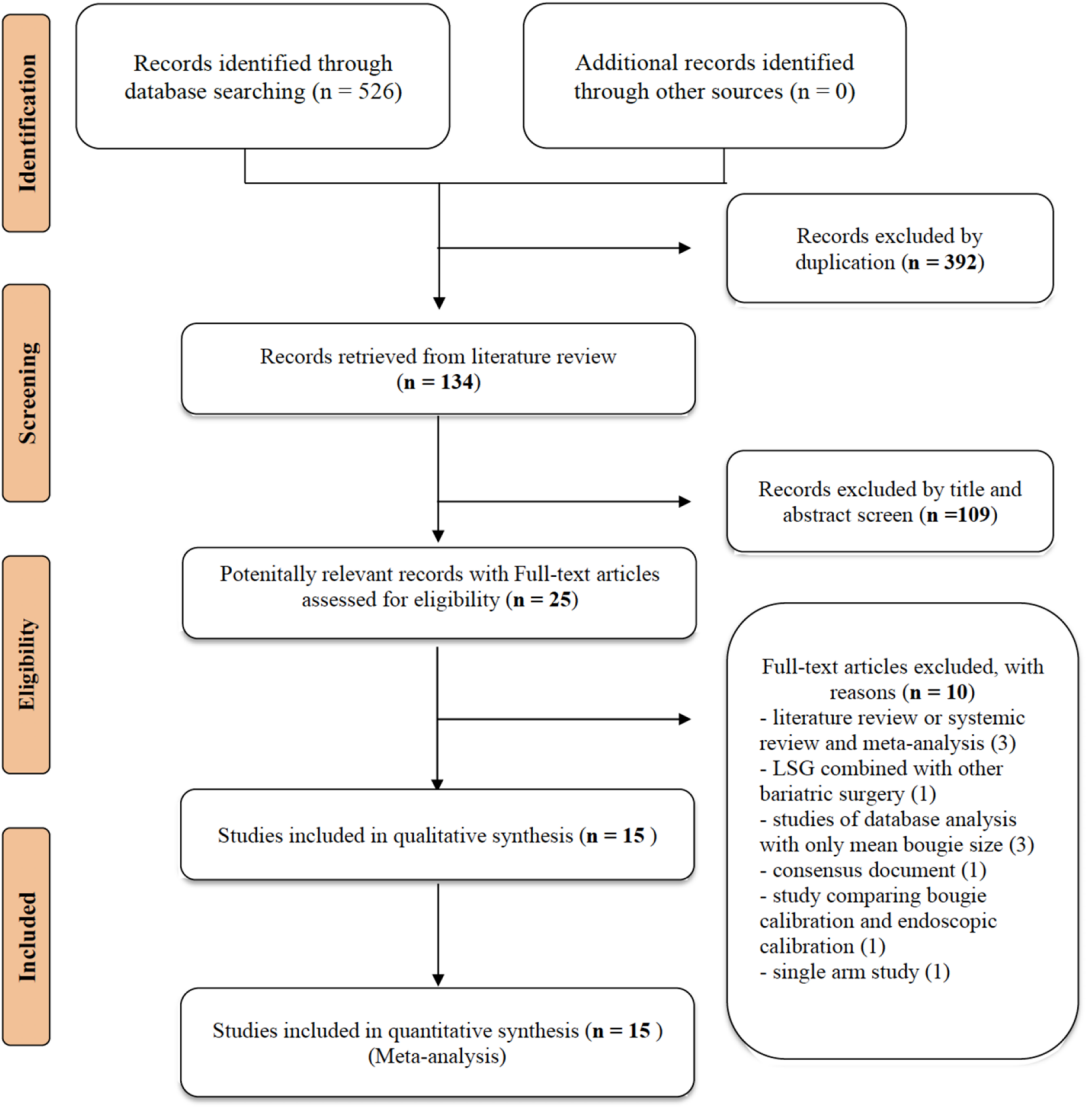
PRISMA flowchart. A total of 15 studies were enrolled in this network meta-analysis. The picture was created with Adobe Photoshop CC 2018 and Sketch Version 45.1 software.

Figure 1 presents the whole flowchart of the current NMA. After the initial screening procedure, a total of 25 articles were considered for full-text review; 10 of which were excluded for various reasons. These 15 trials were included in our study, and a total of 2848 participants receiving LSG using different bougie sizes calibrated as XL, L, M, and S were included.

Study characteristics are summarized in Table 1. The sample size of enrolled studies ranges from 24 to 1395 patients with morbid obesity. The total numbers of RCTs and observational cohort studies are 687 and 2161 patients respectively. Among these participants, the mean age of enrolled patients was 37.46 years. The mean BMI and body weight of participants were 46.97 kg/m^2^ and 121.16 kg, respectively. The bougie calibration sizes ranged from 27 to 60 Fr.

**Table 1:** Characteristic of enrolled studies.

Author year	Design (Country)	Population (Number)	Age (mean)	Male (%)	BMI (mean, kg/m^2^)	Body weight (mean, kg)	Follow up duration (months)	Bougie sizes (Fr., French)	Staple line reinforcement (%)
Braghetto, 2007	PCT (Spain)	Patients with morbid obesity, (n = 50)	38.20 (years)	22	37.9	103.4	6, 12	32/40	100%
Weiner, 2007	PCT (Poland)	Patients with morbid obesity, (n = 120)	40.3 (years)	28.33	60.7	179.8	24	32/44	100%
Parikh, 2008	ReCT (The U.S)	Patients with morbid obesity, (n = 135)	43.5 (years)	NR	60.1	NR	12	40/60	NR
Atkins, 2012	ReCT (Australia)	Patients with morbid obesity, (n = 294)	42.9 (years)	22.67	42.2	119.20	48	40/50	NR
Aldaqal, 2013	RCT (Saudi Arabia)	Patients with morbid obesity, (n = 90)	42.0 (years)	31.75	45.75	NR	12	34/36	NR
Spivak, 2014	RCT (Israel)	Patients with morbid obesity, (n = 120)	41.35 (years)	34.17	43.16	119.45	12	32/42	NR
Ellatif, 2014	ReCT (Egypt)	Patients with morbid obesity, (n = 1395)	33.0 (years)	26.0	46.0	109.0	6,12,24,36, 48,60,72,84	< 36/ > 44	32%
Hawasli, 2015	PCT (The U.S.)	Patients with morbid obesity, (n = 131)	44.72 (years)	NR	47.05	187.2	12	32/36	0%
Seki, 2016	ReCT (Japan)	Patients with morbid obesity, (n = 179)	40.70 (years)	50.30	43.30	120.40	12,24,36,48, 60	36/45	NR
Cal, 2016	RCT (Argentina)	Patients with morbid obesity, (n = 126)	42.05 (years)	14.29	43.91	NR	6,12	27/39	100%
Balla, 2017	ReCT (Italy)	Patients with morbid obesity, (n = 127)	45.02 (years)	24.40	45.54	NR	14.8–69.7*	36/42/48	72%
Hady, 2018	RCT (Poland)	Patients with morbid obesity, (n = 120)	43.15 (years)	36.67	47.47	135.83	6	32/36/40	NR
Helmy, 2018	RCT (Egypt)	Patients with morbid obesity, (n = 60)	35.0 (years)	28.33	46.66	NR	6,12	32/40	NR
Omarov, 2020	RCT (Azerbaijan)	Patients with morbid obesity, (n = 123)	40.84 (years)	26.02	48.20	NR	3,6,12,24	32/36	NR
Abo-Elelaa, 2020	RCT (Egypt)	Patients with morbid obesity, (n = 48)	NR	37.50	48.95	137.86	1,3,6,12	32/40	NR

PCT = prospective cohort study; ReCT = retrospective cohort study; BMI = body mass index; RCT = randomized controlled trial; U.S. = United States; NR = not reported.

*The range of following period.

### Outcome measure: EWL

**Figure 2:**
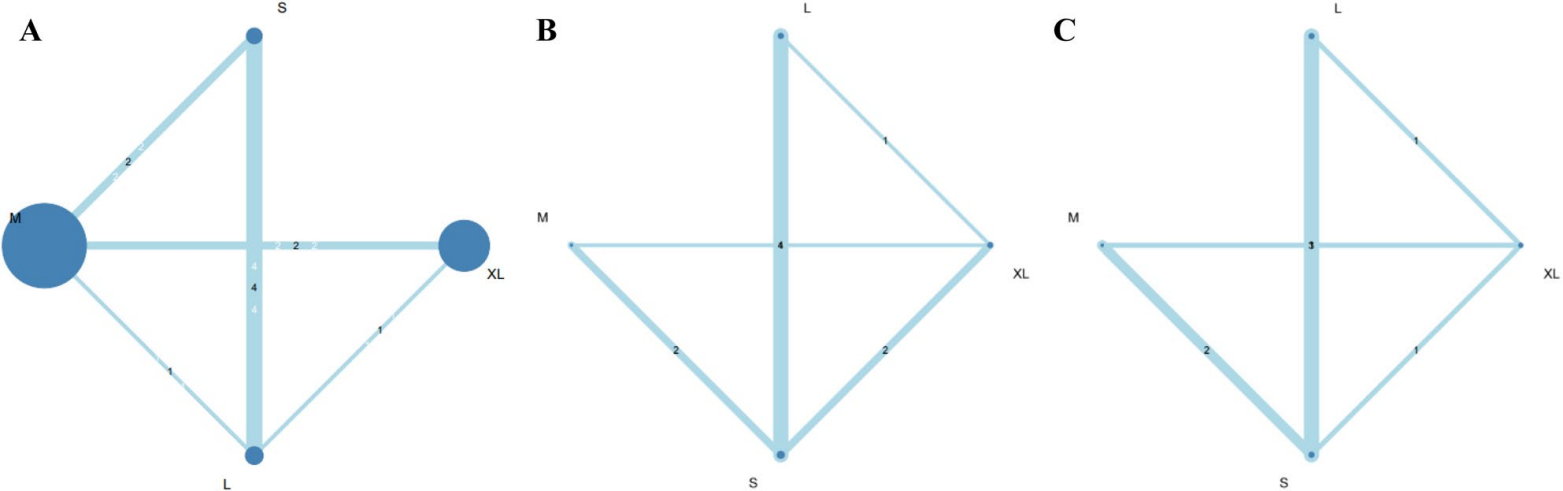
(**A**) Network structure of outcome measure (excess weight loss). Network plot of excess weight loss comparisons. The picture was created with Adobe Photoshop CC 2018 and Sketch Version 45.1 software. (**B**) Network structure of outcome measure (total complications). Network plot of total complication comparisons. The picture was created with Adobe Photoshop CC 2018 and Sketch Version 45.1 software. (**C**) Network structure of outcome measure (staple line leak). Network plot of staple line leak comparisons. The picture was created with Adobe Photoshop CC 2018 and Sketch Version 45.1 software.

**Figure 3:**
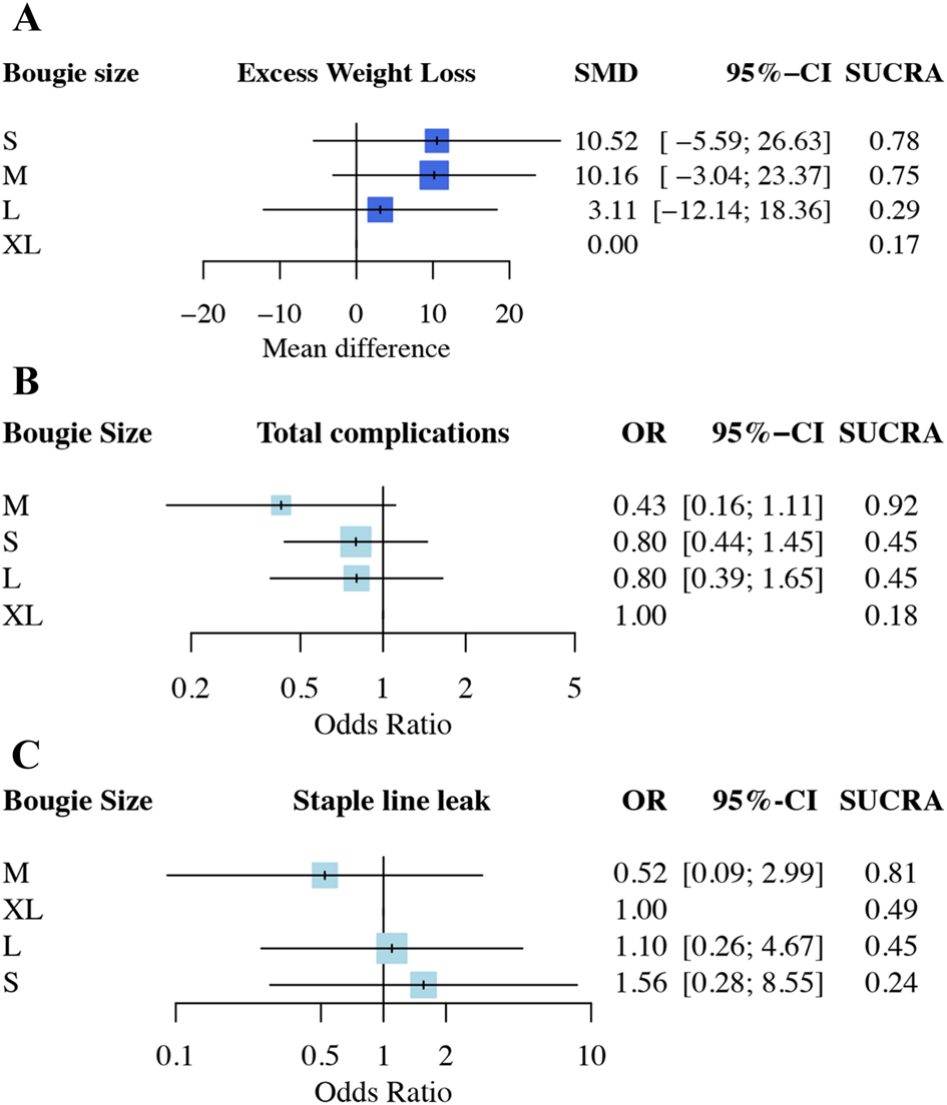
(**A**) Network meta-analysis results of excess weight loss (EWL). SUCRA values among different bougie sizes according to excess weight loss. S- and M-sized bougie had similar effects associated with the greatest EWL improvement among all various bougie sizes. The picture was created with Adobe Photoshop CC 2018 and Sketch Version 45.1 software. (**B**) Network meta-analysis results of total complications. SUCRA values among different bougie sizes according to total complications. M-sized bougie was associated with the lowest incidence of total complications. The picture was created with Adobe Photoshop CC 2018 and Sketch Version 45.1 software. (**C**) Network meta-analysis results of the staple line leak (SLL). SUCRA values among different bougie sizes according to total complications. M-sized bougie was associated with the lowest incidence of SLL. The picture was created with Adobe Photoshop CC 2018 and Sketch Version 45.1 software.

Eight studies (2168 patients; 4 treatment nodes) were found to investigate EWL with different bougie sizes after LSG in the current NMA (the network structure figure is shown in Fig. 2A)^14,18–21,23,24,26^. Figure 3A presents results of EWL percentage. According to the SUCRA value, S- and M-sized bougie had similar effect and was associated with the highest EWL among all different bougie sizes (S-sized: SMD, 10.52; 95% CI − 5.59 to − 26.63; SUCRA, 0.78; M-sized: SMD, 10.16; 95% CI − 3.04 to 23.37; SUCRA, 0.75). Table S5.1 shows head-to-head comparison details of outcomes.

### Outcome measure: total complications

Ten studies (1160 patients; 4 treatment nodes) discussed total complications with different bougie sizes after LSG in the current NMA (the network structure figure is shown in Fig. 2B)^12,13,15,17,19,21,22,24–26^. Figure 3B presents total complications. According to the SUCRA value, M-sized bougie was associated with the lowest incidence of total complications (M-sized: OR, 0.43; 95% CI 0.16–1.11; SUCRA, 0.92). The S- and L-sized bougies had similar side effect incidence in total complication and ranked as second (S-sized: OR, 080; 95% CI 0.44–1.45; SUCRA: 0.45; L-sized: OR, 0.80; 95% CI 0.39–1.65, SUCRA: 0.45). Table S5.2 shows head-to-head comparison details of outcomes.

### Outcome measure: staple line leak

Eight studies (1026 patients; 4 treatment nodes) reported the postoperative SLL in the current NMA (the network structure figure is shown in Fig. 2C)^15,17,19,21,22,24–26^. Figure 3C presents the results of SLL. No significant difference was observed in S-, M-, and L-sized bougies in SLL than that in the XL-sized bougie. According to the SUCRA, the M-sized bougie was associated with the lowest incidence of SLL among all different bougie sizes (M-sized: OR 0.52; 95% CI 0.09–2.99; SUCRA, 0.81). Table S5.3 shows head-to-head comparison details of outcomes.

### Inconsistency and sensitivity analysis

In the EWL outcome, global inconsistency existed with statistical significance between design inconsistency in design-by-treatment interaction model. Local inconsistency was also observed with statistical significance in the node-splitting model.

Thus, a sensitivity analysis was performed to evaluate possible reasons of inconsistency. As heterogeneity may exist in the follow-up period between studies, Hady et al.’s study was excluded with a 6-month follow-up^23^. The remaining seven included studies had constant follow-up period of at least 1 year and enrolled in our sensitivity analysis. In the sensitivity analysis, S-, M-, and L-sized bougies were associated with significantly better EWL than that in the XL-sized. According to the SUCRA value, the S-sized bougie was associated with the greatest EWL among all of the different sizes of bougie. (S-sized: SMD, 7.76; 95% CI 1.75–13.77; SUCRA, 0.84). Furthermore, the L- and M-sized bougies had similar effect and were ranked as second and third, respectively (L-sized: SMD, 6.42; 95% CI 0.52–12.33; SUCRA, 0.59; M-sized: SMD, 5.78; 95% CI 2.29–9.27; SUCRA, 0.56). The sensitivity analysis results showed no network inconsistency (Figure S1).

In outcomes of total complication or SLL, no inconsistency was observed between evidence derived from direct and indirect comparisons, including either global inconsistency as assessed using the design-by-treatment interaction model or local inconsistency as assessed using the node-splitting model (Table S6).

### Risk of bias, inconsistency, and publication bias

We found that 57.1% (100/175 items), 27.4% (48/175 items), and 15.4% (27/175 items) of included studies were assessed as low, unclear, and high risk of bias, respectively. Funding sources and concealing procedure after randomization mainly contributed to the high and unclear risk of bias, respectively (Table S3.1 and Table S3.2).

The assumption of network transitivity was established by visually inspecting tables with patient population across included studies (Table 1). Formal assessments of funnel plots across included studies were conducted for all outcomes and revealed general symmetry without publication bias. Results of the Egger’s test indicated were not statistically significant, which also suggested no publication bias in the present NMA (Figure S2.1 to S2.3).

## Discussion

To the best of our knowledge, this is the first network meta-analysis that analyzes the effectiveness and safe range of bougie sizes to achieve reduced weight loss and lower complications for LSG. The network meta-analysis presented herein not only shows the relative treatment effect (EWL) and associated complications from all pairwise comparisons but also offered ranking of different bougie sizes^34,36,39–42^. We comprehensively reviewed the major database and included only high-quality articles. Based on currently available evidences, our results suggest that S- and M-sized bougies both have the greatest EWL, and the latter was associated with the lowest incidence of total complications, including SLL. Within our network meta-analysis, using the M-sized bougie (bougie size between 33 and 36 Fr., including 36 Fr.) for intraoperative calibration is an optimal choice to balance the effectiveness and safety during LSG.

LSG itself is considered as a purely restrictive bariatric surgical procedure and also has impacts on gastrointestinal motility, hormonal regulations, and gut microbiota. LSG has been demonstrated to increase the rate of gastric emptying and intestinal transit. Studies also found increased glucagon-like peptide 1 and peptide YY levels and increased endocrine functions for bile acids after LSG^9,43,44^. In regard to clinical effects, LSG can produce efficient weight loss and improve obesity-related comorbidities accordingly, such as type 2 diabetes mellitus, hypertension, dyslipidemia, or obstructive sleep apnea^43–45^.

Therefore, an ideal gastric sleeve with the proper size should be created to strike the balance between the acceptable weight loss and occurrence of complications. Intraoperative bougie calibration is an essential part for LSG via different bougie tube sizes to assist bariatric surgeons and to determine the expected gastric tube. Although the clinical significance of an ideal gastric sleeve cannot be overemphasized, the ideal bougie size used in LSG remains to be established. In 2013, a literature review to discuss the ideal bougie size reported the L-sized bougie could decrease the incidence of SLL with the similar EWL effect as the S-sized bougie^46^. Series of RCTs or retrospective studies to compare thinner and bigger size of bougie calibration were conducted in recent decades, and conflict results were presented^12,13,17–22^. In 2018, Wang et al. conducted a meta-analysis that discovered thinner-sized bougie in LSG was more effective in augmenting weight loss, and overall complications were not increased^28^. With new evidences enrolled in this study, our results are in agreement with that of previous meta-analyzes, and we further determined the ideal range of bougie size balancing the effect of weight loss and safety in clinical practice.

In addition to bougie sizes, some studies advocated that related surgical manipulation of the distance from the pylorus with associated antral resection/preservation might influence LSG outcomes^47–50^. For the restrictive purpose, antral resection with shorter distance from the pylorus limits more gastric volume to create a smaller gastric tube and is hypothesized to increase intragastric pressure and decrease the distention ability after eating, which results in early satiety theoretically^47–50^. However, some surgeons suggested antral preservation to prevent possible gastric outlet stenosis and to decrease intragastric pressure in order to reduce the SLL risk^51–54^. In a 2018 meta-analysis, antral resection is associated with better effect of weight loss and without increased risk of surgical complications as compared with antral preservation^54^.

Some adverse events reported to be associated with LSG were bleeding, nausea, wound infection, SLL, or de novo GERD^11,61^. However, the advantages and disadvantages of LSG for GERD remains controversial. In 2019, a systematic literature review found LSG is associated with an increased incidence of de novo GERD. Those with mild GERD might have improved symptoms after LSG; however, patients with morbid obesity, severe reflux and erosive esophagitis may have high possibility of persistent GERD thereafter^55^. Moreover, SLL is a catastrophic complication after LSG, and previous studies have postulated that larger bougie size may decrease the SLL risk^46,56^. Nevertheless, surgeons’ personal experience might play a vital role in decreasing and even preventing this undesirable complication^57^. In 2018, Demusy et al. analyzed the nationwide data in the United States, which disclosed that the bougie size was not associated with postoperative leak rate, and the risk of bleeding and reoperation was decreased via concomitant staple line reinforcement intraoperatively^58^. In our network meta-analysis, overall complication rates or SLL did not significantly increase in the group with bougie size of < 32 Fr. (S-sized group), whereas the M-sized group was associated with the lowest incidence of total complications.

Herein, we have categorized different bougie sizes into four groups for the following reasons. First, we routinely used a 32-Fr. oral gastric tube during vertical gastric sleeve stapling to format the gastric tube in our institution. Accordingly, we choose 32 Fr. as the first cut point. Moreover, we set 36 Fr. as the second cut point because it was found to be the optimal bougie size to augment EWL in recent studies^18,28,59^. A retrospective multicenter cohort study conducted by Sánchez-Santos et al. in 2016 concluded that a bougie size of > 40 Fr. had a protective effect to minimize the overall complication rate^60,61^. Therefore, 40-Fr. bougie was used as the third cut point to investigate the potential protective effect among the group with larger bougie sizes.

Some modifications have been made to increase the strength of the evidence in our network meta-analysis. First, we strictly followed standardized guidelines based on the PRISMA statement to improve reporting of systematic reviews^29,30^. Second, inconsistency and sensitivity analyzes were performed to evaluate possible reasons of inconsistency, and factors that could increase inconsistency were successfully identified and excluded. Nevertheless, the present network meta-analysis has three limitations. First, to increase patient numbers in our studies, some prospective and retrospective cohort studies, which may decrease the strength of the evidence were enrolled. Second, these four groups of different bougie sizes were categorized artificially, which may lead to some potential bias. Third, the criteria of reported complications in enrolled studies may be different, which may result in inaccurate complication rates. Therefore, results of this network meta-analysis should be cautiously interpreted.

## Conclusion

Based on our network meta-analysis and current evidences, S- and M-sized bougies had similar effects of EWL, with the latter being associated with the lowest incidence of total complications, including SLL. Intraoperative calibration with M-sized bougie (33–36 Fr.) is an optimal choice to balance the effectiveness and safety for patients with morbid obesity undergoing LSG.

## Supplementary Information

Supplementary Information.
