# Supplementary material for: Promising effects of 33 to 36 Fr. bougie calibration for laparoscopic sleeve gastrectomy: a systematic review and network meta-analysis
Source: Sci Rep. 2021 Jul 26;11:15217. doi: 10.1038/s41598-021-94716-1 (PMC8313581; doi:10.1038/s41598-021-94716-1)
Supplement: Supplementary file 1 — Supplementary Information. [file 41598_2021_94716_MOESM1_ESM.docx]

**Supporting Information**

**Promising Effects of 33 to 36 Fr. Bougie Calibration for Laparoscopic Sleeve Gastrectomy: A Systematic Review and Network Meta-Analysis**

Po-Chih Chang^1,2,3,4^, Kai-Hua Chen^5#^, Hong-Jie Jhou^6,7^, Po-Huang Chen^8+^, Chih-Kun Huang^9^, Cho-Hao Lee^10^, Ting-Wei Chang^5*^

**Contents**

Table S1. PRISMA Checklist and MOOSE Checklist

Table S2. Search strategy

Table S3. Assessment of risk of bias

Table S4. Outcome definitions of enrolled studies

Table S5. Results the head-to-head comparison of network meta-analysis

Table S6. Estimation of inconsistency

Figure S1. Sensitivity Analyses

Figure S2. Comparison-adjusted funnel plots and Egger’s test

**Table S1** PRISMA Checklist and MOOSE Checklist

**Table S1.1** PRIMSA checklist

| **Section/**  **Topic** | **Item** | **Checklist Item** | **Reported on Page #** |
| --- | --- | --- | --- |
| **TITLE** | | | |
| Title | 1 | Identify the report as a systematic review *incorporating a network meta-analysis (or related form of meta-analysis).* | 1 |
| **ABSTRACT** | | | |
| Structured summary | 2 | Provide a structured summary including, as applicable:  **Background:** main objectives  **Methods:** data sources; study eligibility criteria, participants, and interventions; study appraisal; and *synthesis methods, such as network meta-analysis.*  **Results:** number of studies and participants identified; summary estimates with corresponding confidence/credible intervals; *treatment rankings may also be discussed. Authors may choose to summarize pairwise comparisons against a chosen treatment included in their analyses for brevity.*  **Discussion/Conclusions:** limitations; conclusions and implications of findings.  **Other:** primary source of funding; systematic review registration number with registry name. | 3 |
| **INTRODUCTION** | | | |
| Rationale | 3 | Describe the rationale for the review in the context of what is already known*, including mention of why a network meta-analysis has been conducted.* | 5-6 |
| Objectives | 4 | Provide an explicit statement of questions being addressed, with reference to participants, interventions, comparisons, outcomes, and study design (PICOS). | 5-6 |
| **METHODS** |  |  |  |
| Protocol and registration | 5 | Indicate whether a review protocol exists and if and where it can be accessed (e.g., Web address); and, if available, provide registration information, including registration number. | 7 |
| Eligibility criteria | 6 | Specify study characteristics (e.g., PICOS, length of follow-up) and report characteristics (e.g., years considered, language, publication status) used as criteria for eligibility, giving rationale. *Clearly describe eligible treatments included in the treatment network, and note whether any have been clustered or merged into the same node (with justification).* | 7-8 |
| Information sources | 7 | Describe all information sources (e.g., databases with dates of coverage, contact with study authors to identify additional studies) in the search and date last searched. | 7 |
| Search | 8 | Present full electronic search strategy for at least one database, including any limits used, such that it could be repeated. | 7,  Supplementary Information 2 |
| Study selection | 9 | State the process for selecting studies (i.e., screening, eligibility, included in systematic review, and, if applicable, included in the meta-analysis). | 7-8 |
| Data collection process | 10 | Describe method of data extraction from reports (e.g., piloted forms, independently, in duplicate) and any processes for obtaining and confirming data from investigators. | 8-9 |
| Data items | 11 | List and define all variables for which data were sought (e.g., PICOS, funding sources) and any assumptions and simplifications made. | 9-11 |
| Geometry of the network | S1 | Describe methods used to explore the geometry of the treatment network under study and potential biases related to it. This should include how the evidence base has been graphically summarized for presentation, and what characteristics were compiled and used to describe the evidence base to readers. | 11 |
| Risk of bias within individual studies | 12 | Describe methods used for assessing risk of bias of individual studies (including specification of whether this was done at the study or outcome level), and how this information is to be used in any data synthesis. | 11 |
| Summary measures | 13 | State the principal summary measures (e.g., risk ratio, difference in means). *Also describe the use of additional summary measures assessed, such as treatment rankings and surface under the cumulative ranking curve (SUCRA) values, as well as modified approaches used to present summary findings from meta-analyses.* | 11 |
| Planned methods of analysis | 14 | Describe the methods of handling data and combining results of studies for each network meta-analysis. This should include, but not be limited to:  *Handling of multi-arm trials;*  *Selection of variance structure;*  *Selection of prior distributions in Bayesian analyses; and Assessment of model fit.* | 11 |
| Assessment of Inconsistency | S2 | Describe the statistical methods used to evaluate the agreement of direct and indirect evidence in the treatment network(s) studied. Describe efforts taken to address its presence when found. | 11 |
| Risk of bias across studies | 15 | Specify any assessment of risk of bias that may affect the cumulative evidence (e.g., publication bias, selective reporting within studies). | 11 |
| Additional analyses | 16 | Describe methods of additional analyses if done, indicating which were pre-specified. This may include, but not be limited to, the following:  Sensitivity or subgroup analyses;  Meta-regression analyses;  *Alternative formulations of the treatment network; and Use of alternative prior distributions for Bayesian analyses (if applicable).* | 11 |
| **RESULTS†** |  |  |  |
| Study selection | 17 | Give numbers of studies screened, assessed for eligibility, and included in the review, with reasons for exclusions at each stage, ideally with a flow diagram. | 12,  Figure 1 |
| Presentation of network structure | S3 | Provide a network graph of the included studies to enable visualization of the geometry of the treatment network. | Figure 2 |
| Summary of network geometry | S4 | Provide a brief overview of characteristics of the treatment network. This may include commentary on the abundance of trials and randomized patients for the different interventions and pairwise comparisons in the network, gaps of evidence in the treatment network, and potential biases reflected by the network structure. | 12-13, Supplementary Information 5 |
| Study characteristics | 18 | For each study, present characteristics for which data were extracted (e.g., study size, PICOS, follow-up period) and provide the citations. | 12, Supplementary Information 2 |
| Risk of bias within studies | 19 | Present data on risk of bias of each study and, if available, any outcome level assessment. | Supplementary Information 3 |
| Results of individual studies | 20 | For all outcomes considered (benefits or harms), present, for each study: 1) simple summary data for each intervention group, and 2) effect estimates and confidence intervals. *Modified approaches may be needed to deal with information from larger networks.* | 12-13 |
| Synthesis of results | 21 | Present results of each meta-analysis done, including confidence/credible intervals. *In larger networks, authors may focus on comparisons versus a particular comparator (e.g. placebo or standard care), with full findings presented in an appendix. League tables and forest plots may be considered to summarize pairwise comparisons.* If additional summary measures were explored (such as treatment rankings), these should also be presented. | 12-14, Supplementary Information 7-8 |
| Exploration for inconsistency | S5 | Describe results from investigations of inconsistency. This may include such information as measures of model fit to compare consistency and inconsistency models, *P* values from statistical tests, or summary of inconsistency estimates from different parts of the treatment network. | 14-15, Supplementary Information 6 |
| Risk of bias across studies | 22 | Present results of any assessment of risk of bias across studies for the evidence base being studied. | 14-15 |
| Results of additional analyses | 23 | Give results of additional analyses, if done (e.g., sensitivity or subgroup analyses, meta-regression analyses*, alternative network geometries studied, alternative choice of prior distributions for Bayesian analyses,* and so forth). | 15-16 |
| **DISCUSSION** | | | |
| Summary of evidence | 24 | Summarize the main findings, including the strength of evidence for each main outcome; consider their relevance to key groups (e.g., healthcare providers, users, and policy-makers). | 17 |
| Limitations | 25 | Discuss limitations at study and outcome level (e.g., risk of bias), and at review level (e.g., incomplete retrieval of identified research, reporting bias). *Comment on the validity of the assumptions, such as transitivity and consistency. Comment on any concerns regarding network geometry (e.g., avoidance of certain comparisons).* | 21 |
| Conclusions | 26 | Provide a general interpretation of the results in the context of other evidence, and implications for future research. | 7 |
| **FUNDING** | | | |
| Funding | 27 | Describe sources of funding for the systematic review and other support (e.g., supply of data); role of funders for the systematic review. This should also include information regarding whether funding has been received from manufacturers of treatments in the network and/or whether some of the authors are content experts with professional conflicts of interest that could affect use of treatments in the network. | 22 |

PICOS = population, intervention, comparators, outcomes, study design.

* Text in italics indicateS wording specific to reporting of network meta-analyses that has been added to guidance from the PRISMA statement.

**Table S1.2** MOOSE Checklist

| **Item No** | **Recommendation** | **Reported on Page No** |
| --- | --- | --- |
| Reporting of background should include | | |
| 1 | Problem definition | 5-6 |
| 2 | Hypothesis statement | 5-6 |
| 3 | Description of study outcome(s) | 5-6 |
| 4 | Type of exposure or intervention used | 5-6 |
| 5 | Type of study designs used | 5-6 |
| 6 | Study population | 8 |
| Reporting of search strategy should include | | |
| 7 | Qualifications of searchers (eg, librarians and investigators) | 8-9 |
| 8 | Search strategy, including time period included in the synthesis and keywords | 7 |
| 9 | Effort to include all available studies, including contact with authors | 7-9 |
| 10 | Databases and registries searched | 7 |
| 11 | Search software used, name and version, including special features used (eg, explosion) | Manual |
| 12 | Use of hand searching (eg, reference lists of obtained articles) | 7 |
| 13 | List of citations located and those excluded, including justification | 7 |
| 14 | Method of addressing articles published in languages other than English | 7 |
| 15 | Method of handling abstracts and unpublished studies | 7-8 |
| 16 | Description of any contact with authors | 7 |
| Reporting of methods should include | | |
| 17 | Description of relevance or appropriateness of studies assembled for assessing the hypothesis to be tested | 6 |
| 18 | Rationale for the selection and coding of data (eg, sound clinical principles or convenience) | Appendices |
| 19 | Documentation of how data were classified and coded (eg, multiple raters, blinding and interrater reliability) | 8-10 |
| 20 | Assessment of confounding (eg, comparability of cases and controls in studies where appropriate) | 8-10 |
| 21 | Assessment of study quality, including blinding of quality assessors, stratification or regression on possible predictors of study results | 8-10 |
| 22 | Assessment of heterogeneity | 10-11 |
| 23 | Description of statistical methods (eg, complete description of fixed or random effects models, justification of whether the chosen models account for predictors of study results, dose-response models, or cumulative meta-analysis) in sufficient detail to be replicated | 10-11 |
| 24 | Provision of appropriate tables and graphics | Tables  Figs 1-3 |

| **Item No** | **Recommendation** | **Reported on Page No** |
| --- | --- | --- |
| Reporting of results should include | | |
| 25 | Graphic summarizing individual study estimates and overall estimate | Table 1, Fig 1 |
| 26 | Table giving descriptive information for each study included | Table 1 |
| 27 | Results of sensitivity testing (eg, subgroup analysis) | 14-15 |
| 28 | Indication of statistical uncertainty of findings | 14-15 |
| Reporting of discussion should include | | |
| 29 | Quantitative assessment of bias (eg, publication bias) | 15-16 |
| 30 | Justification for exclusion (eg, exclusion of non-English language citations) | 7 |
| 31 | Assessment of quality of included studies | Appendices |
| Reporting of conclusions should include | | |
| 32 | Consideration of alternative explanations for observed results | 17 |
| 33 | Generalization of the conclusions (ie, appropriate for the data presented and within the domain of the literature review) | 22 |
| 34 | Guidelines for future research | 22 |
| 35 | Disclosure of funding source | 23 |

From: Stroup DF, Berlin JA, Morton SC, et al, for the Meta-analysis Of Observational Studies in Epidemiology (MOOSE) Group. Meta-analysis of Observational Studies in Epidemiology. A Proposal for Reporting. *JAMA*. 2000;283(15):2008-2012. doi: 10.1001/jama.283.15.2008.

**Table** **S2** Search Strategy

**Table S2** Search Strategy

| **Search terms** | Laparoscopic sleeve gastrectomy and bougie calibration or bougie size |
| --- | --- |
| **PubMed** | ((((((((("laparoscopes"[MeSH Terms] OR "laparoscopes"[All Fields]) OR "laparoscope"[All Fields]) OR "laparoscopical"[All Fields]) OR "laparoscopically"[All Fields]) OR "laparoscopics"[All Fields]) OR "laparoscopy"[MeSH Terms]) OR "laparoscopy"[All Fields]) OR "laparoscopic"[All Fields]) AND ((("sleeve"[All Fields] OR "sleeved"[All Fields]) OR "sleeves"[All Fields]) OR "sleeving"[All Fields]) AND (("gastrectomy"[MeSH Terms] OR "gastrectomy"[All Fields]) OR "gastrectomies"[All Fields]) AND ("bougie"[All Fields] OR "bougies"[All Fields]) AND (((((((((("calibrant"[All Fields] OR "calibrants"[All Fields]) OR "calibrate"[All Fields]) OR "calibrated"[All Fields]) OR "calibrates"[All Fields]) OR "calibrating"[All Fields]) OR "calibration"[MeSH Terms]) OR "calibration"[All Fields]) OR "calibrations"[All Fields]) OR "calibrator"[All Fields]) OR "calibrators"[All Fields])) OR (("bougie"[All Fields] OR "bougies"[All Fields]) AND "size"[All Fields]) |
| **Embase** | ('laparoscopic sleeve gastrectomy'/exp OR 'laparoscopic sleeve gastrectomy') AND ('bougie calibration' OR (('bougie'/exp OR bougie) AND ('calibration'/exp OR calibration))) OR 'bougie size' OR (('bougie'/exp OR bougie) AND ('size'/exp OR size)) |

**Table S3** Assessment of risk of bias

| **Table S3.1** Risk of bias in the included randomized controlled trial studies. | | | | | | | |
| --- | --- | --- | --- | --- | --- | --- | --- |
| First author, year | Random Sequence generation (Selection bias) | Allocation Concealment (Selection bias) | Blinding of participants and personnel (Performance bias) | Blinding of outcome assessment (Detection bias) | Incomplete outcome data (Attrition bias) | Selective reporting (Reporting bias) | Other bias |
| Aldaqal, 2013 | Low | Low | High | High | Low | Unclear | Low |
| Spivak, 2014 | Low | Unclear | High | High | Low | Low | Low |
| Cal, 2016 | Low | Unclear | High | High | Low | Low | Low |
| Hady, 2018 | Low | Unclear | High | High | Low | Low | Low |
| Helmy, 2018 | Low | Low | High | High | Low | Unclear | Low |
| Omarov, 2020 | Low | Low | High | High | Low | Low | Low |
| Abo-Elelaa, 2020 | Low | Unclear | High | High | Low | Unclaer | Low |
|  |  |  |  |  |  |  |  |
|  |  |  |  |  |  |  |  |

| **Table S3.2** Newcastle-Ottawa Scale quality assessment scale for cohort studies | | | | | | | | |
| --- | --- | --- | --- | --- | --- | --- | --- | --- |
| First author, year | Representativeness of the exposed cohort | Selection of  the nonexposed cohort | Ascertainment of exposure | Demonstration that outcome of interest was not present at start of study | Comparability of cohorts on  the basis of  the design  or analysis | Assessment of outcome | Was follow-up long enough for outcomes to occur | Adequacy of follow up of cohorts |
| Braghetto, 2007 | **🟑** | **🟑** | **🟑** | **🟑** | **🟑** | **🟑** | **🟑** | **🟑** |
| Weiner,2007 | **🟑** | **🟑** | **🟑** | **🟑** | **🟑** | **🟑** | **🟑** | **🟑** |
| Parikh,2008 | **🟑** | **🟑** | **🟑** | **🟑** | **🟑🟑** | **🟑** | **🟑** | **🟑** |
| Atkins,2012 | **🟑** | **🟑** | **🟑** | **🟑** | **🟑** | **🟑** | **🟑** | **🟑** |
| Ellatif, 2014 | **🟑** |  | **🟑** | **🟑** | **🟑** | **🟑** | **🟑** |  |
| Hawasli,2015 | **🟑** | **🟑** | **🟑** | **🟑** | **🟑** | **🟑** | **🟑** | **🟑** |
| Seki, 2016 | **🟑** | **🟑** | **🟑** | **🟑** | **🟑** | **🟑** | **🟑** |  |
| Balla, 2017 | **🟑** | **🟑** | **🟑** | **🟑** | **🟑🟑** | **🟑** | **🟑** |  |
|  |  |  |  |  |  |  |  |  |
|  |  |  |  |  |  |  |  |  |

**Table S4** Outcome definitions of enrolled stidies

**Table S4** Outcome definitions of enrolled stidies. This table presented the definitions of percentage of excess weight loss, overall complications and gastrointestinal leakage.

| First author, year | Percentage of excess weight loss(%EWL) | Overall complications | Gastrointestinal leakage |
| --- | --- | --- | --- |
| Abo-Elelaa, 2020 | Not mentioned | Bleeding, leakage, acute paraesophageal intrathoracic migration of the sleeve, splenic infarction, intra-abdominal sepsis, wound infection | Not mentioned |
| Omarov, 2020 | No record of this parameter. | Staple line leak, staple line bleeding | Not mentioned |
| Helmy, 2020 | (Preoperative weight-Followup weight)/ (Preoperative weight-ideal body weight)x100 | Postoperative bleeding, postoperative leak, postoperative vomiting, portside hematoma. | Gastrogrifin swallow exam |
| Hady, 2018 | %EWL=(preoperative weightfollow-up weight)/(preoper-  ative weightideal weight)100  For ideal weight calculations, the Lorenz formulas were used:  Ideal female weight=(height in cm100)((height in  cm150)/2),  Ideal male weight=(height in cm100)((height cm  150)/4). | Acute pancreatitis, trocar site bleeding, superficial thrombophlebitis. | Not mentioned |
| Balla, 2017 | Not mentioned. | Application of Clavien-Dindo classification, leakage, leeding, spleen hematoma. | Leaks were classified  according to the International Expert Panel Consensus Statement. |
| Seki, 2016 | Not mentioned. | Postoperative leaks, postoperative bleeding, sleeve stenosis, acute renal failure , Intractable GERD, Bleeding due to severe GERD, Repeated hypoglycemia | Not mentioned. |
| Cal, 2016 | Not mentioned | Postoperative leak | Not mentioned. |
| Hawasli, 2015 | Not mentioned | Postoperative leak, nausea and dehydration, wound infection, wound seroma, reflux, nonspecific abdominal pain, mesenteric vein thrombosis, leg pain. | Not mentioned. |
| Spivak, 2014 | Not mentioned. | Portal vein thrombosis, postoperative bleeding, nausea and dehydration, nonspecific abdominal pain, deteriorated GERD, splenic bleeding. | Not mentioned. |
| Ellatif, 2014 | Not mentioned. | Postoperative leak, sepsis, vomiting, nausea, dehydration, GERD | Gastrograffin study |
| Aldaqal, 2013 | Not mentioned. | Postoperative leak, postoperative bleeding | Not mentioned. |
| Atkins, 2012 | (preoperative weight – current weight)/ (preoperative weight – ideal weight)x100 | Postoperative leak, gastric fistula, postoperative bleeding, Infected perigastric haematoma | Not mentioned. |
| Braghetto, 2007 | No record of this parameter. | Postopertive bleeding | GI dtudy |
| Weiner, 2007 | No record of this parameter. | Staple line leak, wound infection, postoperative bleeding, GERD, constipation, vomiting, gastric pain, diarrhea. | Methylene-blue test. |

**Table** **S5** Results the head-to-head comparison of network meta-analysis

**Table S5.1** Excess weight loss

| Outcome: Excess weight loss | | | |
| --- | --- | --- | --- |
| Extra-large | 6.50 (-14.83 to 27.83) | 8.43 (-6.81 to 23.68) | - |
| 3.11 (-12.14 to 18.36) | Large | -18.34 (-29.12 to 2.44) | -7.62 (-18.01 to 2.78) |
| 10.16 (-3.04 to 23.37) | -7.05 (-20.46 to 6.35) | Median | -3.42 (-18.42 to 11.58) |
| 10.52 (-5.59 to 26.63) | -7.41 (-17.34 to 2.52) | -0.36 (-13.40 to 12.68) | Small |
| Network Meta-analysis Estimate Comparator Direct Estimate | | | |

**Table S5.1** Outcomes for excess weight loss in Network Meta-analysis: Head-to-head comparisons

Data are presented as the odds ratio with 95% CI in the column-defining treatment compared with the row-defining treatment. Comparisons should be read from left to right.

**Table S5.2** Total complications

| Outcome: Total complications | | | |
| --- | --- | --- | --- |
| Extra-large | 1.25 (0.49 to 3.17) | 0.38 (0.08 to 1.83) | 0.63 (0.30 to 1.29) |
| 0.80 (0.39 to 1.65) | Large | - | 0.65 (0.26 to 1.64) |
| 0.43 (0.16 to 1.11) | 1.88 (0.63 to 5.59) | Median | 0.56 (0.20 to 1.57) |
| 0.80 (0.44 to 1.45) | 1.01 (0.49 to 2.07) | 0.53 (0.22 to 1.29) | Small |
| Network Meta-analysis Estimate Comparator Direct Estimate | | | |

**Table S5**.2 Outcomes for total complications in Network Meta-analysis: Head-to-head comparisons

Data are presented as the odds ratio with 95% CI in the column-defining treatment compared with the row-defining treatment. Comparisons should be read from left to right.

**Table S5.3** Staple line leak

| Outcome: Staple line leak | | | |
| --- | --- | --- | --- |
| Extra-large | 1.44 (0.28 to 7.58) | 0.35 (0.04 to 3.09) | 1.22 (0.02 to 62.51) |
| 1.10 (0.26 to 4.67) | Large | - | 0.51 (0.09 to 3.03) |
| 0.52 (0.09 to 2.99) | 2.10 (0.31 to 14.48) | Median | 0.48 (0.06 to 3.76) |
| 1.56 (0.28 to 8.55) | 0.70 (0.15 to 3.20) | 0.33 (0.06 to 1.83) | Small |
| Network Meta-analysis Estimate Comparator Direct Estimate | | | |

**Table S5.3** Outcomes for gastrointestinal leakage in Network Meta-analysis: Head-to-head comparisons
Data are presented as the odds ratio with 95% CI in the column-defining treatment compared with the row-defining treatment. Comparisons should be read from left to right.

**Table S6** Estimation of inconsistency

p-value: The p-value is set as 0.05. If the p-value is more than 0.05, the null hypothesis cannot be rejected and the consistency assumption could be accepted. Thus, if the p-value is less than 0.05, which means statistically significant that inconsistency exists.

**Table S6.1** Estimation of inconsistency in outcome for excess weight loss (EWL)

*Design-by-treatment interaction model*

Q statistics to assess homogeneity / consistency

|  | Q | df | p-value |
| --- | --- | --- | --- |
| Total | 92.77 | 6 | <0.0001 |
| Within designs | 5.34 | 3 | 0.1485 |
| Between designs | 87.43 | 3 | <0.0001 |

Design-specific decomposition of within-designs Q statistic

| Design | Q | df | p-value |
| --- | --- | --- | --- |
| L:S | 3.53 | 2 | 0.1712 |
| M:XL | 1.81 | 1 | 0.1785 |

Between-designs Q statistic after detaching of single designs

| Design | Q | df | p-value |
| --- | --- | --- | --- |
| L:S | 22.14 | 2 | <0.0001 |
| L:XL | 78.69 | 2 | <0.0001 |
| M:S | 85.90 | 2 | <0.0001 |
| M:XL | 78.69 | 2 | <0.0001 |
| L:M:S | 0.00 | 1 | 0.9837 |

Q statistic to assess consistency under the assumption of a full design-by-treatment interaction random effects model

|  | Q | df | p-value | tau.within | tau^2^.within |
| --- | --- | --- | --- | --- | --- |
| Between designs | 34.07 | 3 | <0.0001 | 2.5236 | 6.3685 |

*Node-splitting model*

Back-calculation method to split direct and indirect evidence

Fixed effect model:


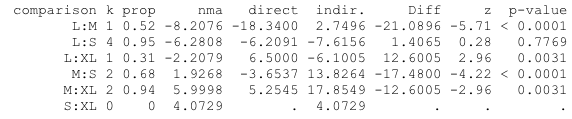


Random effects model:


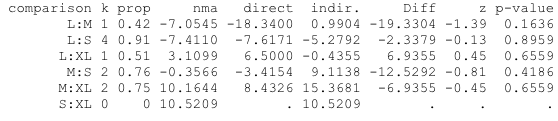


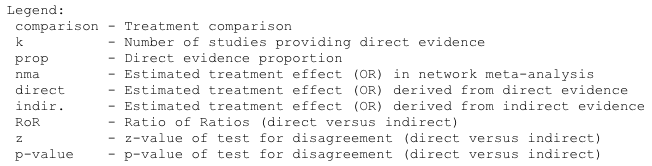


**Table S6.2** Estimation of inconsistency in outcome for total complications

*Design-by-treatment interaction model*

Q statistics to assess homogeneity / consistency

|  | Q | df | p-value |
| --- | --- | --- | --- |
| Total | 5.53 | 7 | 0.5951 |
| Within designs | 3.34 | 5 | 0.6480 |
| Between designs | 2.20 | 2 | 0.3336 |

Design-specific decomposition of within-designs Q statistic

| Design | Q | df | p-value |
| --- | --- | --- | --- |
| L:S | 3.26 | 3 | 0.3538 |
| M:S | 0.01 | 1 | 0.9302 |
| S:XL | 0.07 | 1 | 0.7850 |

Between-designs Q statistic after detaching of single designs

| Design | Q | df | p-value |
| --- | --- | --- | --- |
| L:S | 0.00 | 1 | 0.9351 |
| L:XL | 0.00 | 1 | 0.9351 |
| M:S | 2.16 | 1 | 0.1416 |
| M:XL | 2.16 | 1 | 0.1416 |
| S:XL | 0.81 | 1 | 0.3692 |

Q statistic to assess consistency under the assumption of a full design-by-treatment interaction random effects model

|  | Q | df | p-value | tau.within | tau^2^.within |
| --- | --- | --- | --- | --- | --- |
| Between designs | 2.20 | 2 | 0.3336 | 0 | 0 |

*Node-splitting model*

Back-calculation method to split direct and indirect evidence

Fixed effect model:


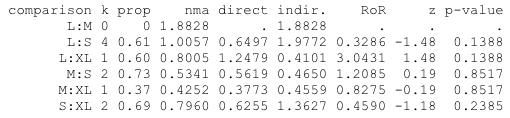


Random effects model:


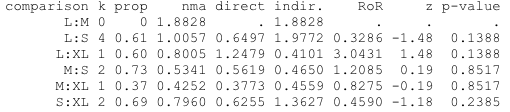


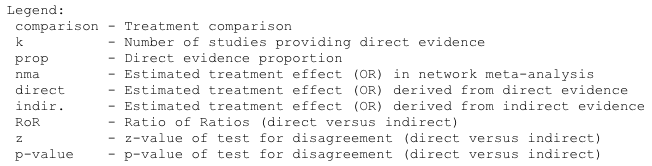


**Table S6.3** Estimation of inconsistency in outcome for staple line leak

*Design-by-treatment interaction model*

Q statistics to assess homogeneity / consistency

|  | Q | df | p-value |
| --- | --- | --- | --- |
| Total | 1.26 | 5 | 0.9390 |
| Within designs | 0.77 | 3 | 0.8556 |
| Between designs | 0.49 | 2 | 0.7844 |

Design-specific decomposition of within-designs Q statistic

| Design | Q | df | p-value |
| --- | --- | --- | --- |
| L:S | 0.40 | 2 | 0.8183 |
| M:S | 0.37 | 1 | 0.5413 |

Between-designs Q statistic after detaching of single designs

| Design | Q | df | p-value |
| --- | --- | --- | --- |
| L:S | 0.04 | 1 | 0.8394 |
| L:XL | 0.04 | 1 | 0.8394 |
| M:S | 0.12 | 1 | 0.7237 |
| M:XL | 0.12 | 1 | 0.7237 |
| S:XL | 0.47 | 1 | 0.4942 |

Q statistic to assess consistency under the assumption of a full design-by-treatment interaction random effects model

|  | Q | df | p-value | tau.within | tau^2^.within |
| --- | --- | --- | --- | --- | --- |
| Between designs | 0.49 | 2 | 0.7844 | 0 | 0 |

*Node-splitting model*

Back-calculation method to split direct and indirect evidence

Fixed effect model:


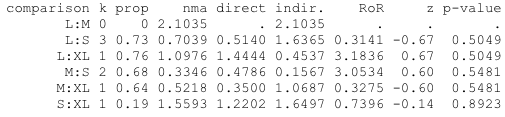


Random effects model:


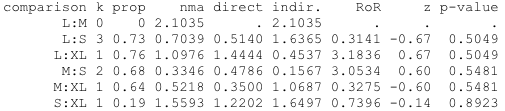


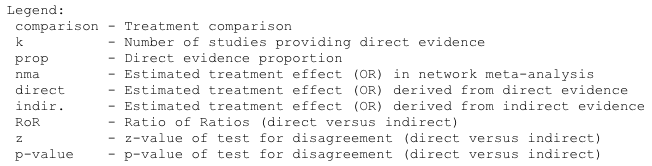


**Figure S1** Sensitivity analyses
Sensitivity Analyses of the studies had constant follow-up period of 1 year
*Excess weight loss (EWL)*


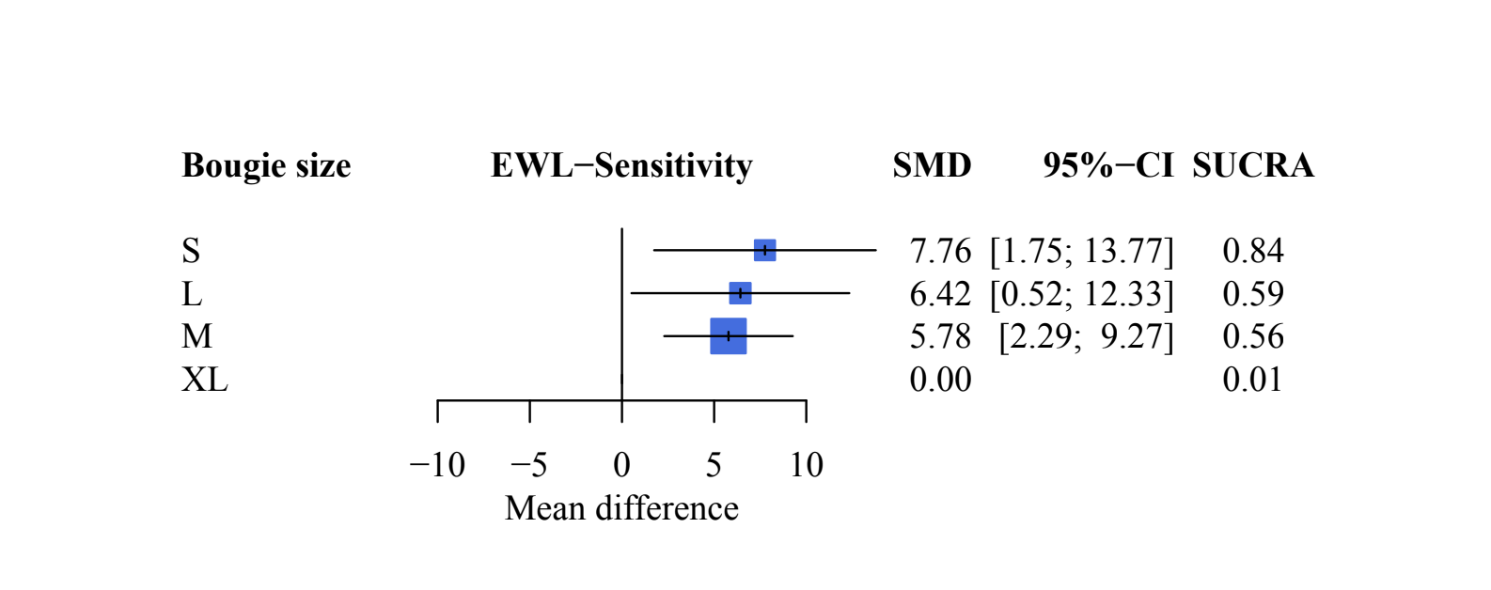


**Figure S1** Sensitivity analyses of the studies had constant follow-up period of 1 year for Excess weight loss:

**XL**, extra-large, defined as the bougie size is larger than 40 Fr.; **L**, large, defined as the bougie size is between 36 and 40 Fr., including 40 Fr.; **M**, median, defined as the bougie size is between 33 and 36 Fr., including 36 Fr.; **S**, small, defined as the bougie size is smaller than 32 Fr., including 32 Fr.; SMD, Standardized mean difference; CI, Conﬁdence interval; SUCRA, Surface under the cumulative ranking curve

**Figure S2** Comparison-adjusted funnel plots and Egger’s test

Excess weight loss


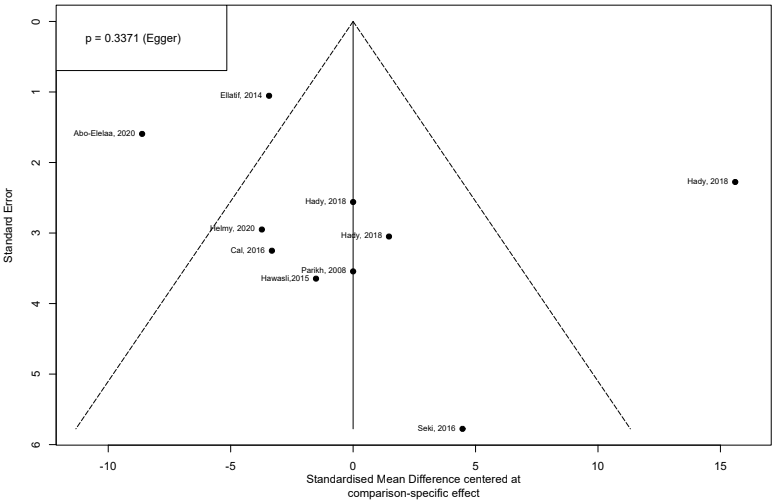


**Figure S2.1** Comparison-adjusted funnel plot in outcome for excess weight loss

Total complications


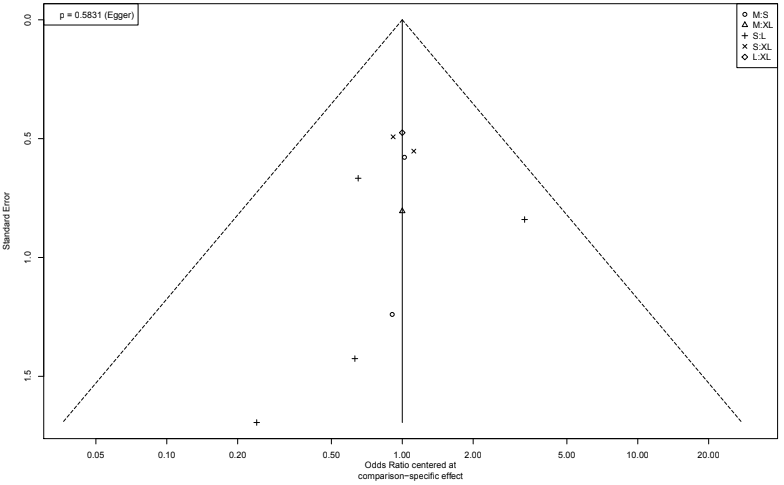


**Figure S2.2** Comparison-adjusted funnel plot in outcome for total complications

Staple line leak


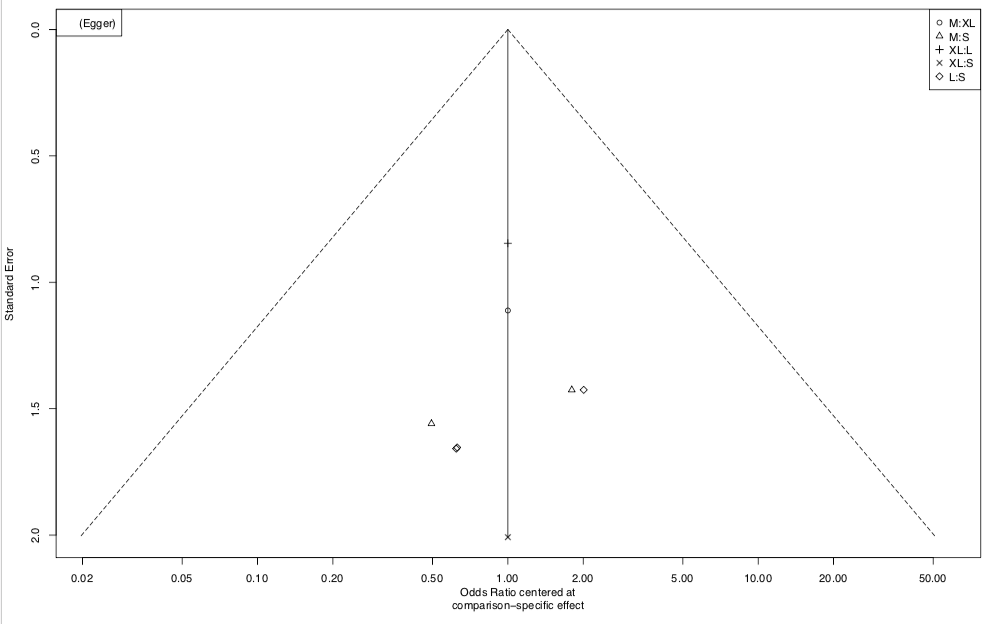


Not applicable

**Figure S2.3** Comparison-adjusted funnel plot in outcome for staple line leak
